# California Wildfires of 2008: Coarse and Fine Particulate Matter Toxicity

**DOI:** 10.1289/ehp.0800166

**Published:** 2009-02-02

**Authors:** Teresa C. Wegesser, Kent E. Pinkerton, Jerold A. Last

**Affiliations:** 1 Department of Pulmonary and Critical Care Medicine and; 2 Department of Pediatrics, School of Medicine, University of California, Davis, California, USA

**Keywords:** air pollution, alveolar macrophage, lung inflammation, mouse, PM_2.5_, PM_10_, source-specific particulate matter

## Abstract

**Background:**

During the last week of June 2008, central and northern California experienced thousands of forest and brush fires, giving rise to a week of severe fire-related particulate air pollution throughout the region. California experienced PM_10–2.5_ (particulate matter with mass median aerodynamic diameter > 2.5 μm to < 10 μm; coarse ) and PM_2.5_ (particulate matter with mass median aerodynamic diameter < 2.5 μm; fine) concentrations greatly in excess of the air quality standards and among the highest values reported at these stations since data have been collected.

**Objectives:**

These observations prompt a number of questions about the health impact of exposure to elevated levels of PM_10–2.5_ and PM_2.5_ and about the specific toxicity of PM arising from wildfires in this region.

**Methods:**

Toxicity of PM_10–2.5_ and PM_2.5_ obtained during the time of peak concentrations of smoke in the air was determined with a mouse bioassay and compared with PM samples collected under normal conditions from the region during the month of June 2007.

**Results:**

Concentrations of PM were not only higher during the wildfire episodes, but the PM was much more toxic to the lung on an equal weight basis than was PM collected from normal ambient air in the region. Toxicity was manifested as increased neutrophils and protein in lung lavage and by histologic indicators of increased cell influx and edema in the lung.

**Conclusions:**

We conclude that the wildfire PM contains chemical components toxic to the lung, especially to alveolar macrophages, and they are more toxic to the lung than equal doses of PM collected from ambient air from the same region during a comparable season.

During the last week of June 2008, central and northern California experienced a major outbreak of wildfires caused by a series of lightning strikes that was unprecedented in the past century in its extent and severity, with transport of smoke over large distances from the fires, especially in the Central Valley. A regional map with the location of the largest of these fires illustrated is available from the California Department of Forestry and Fire Protection (2008). Air quality in the region was severely affected by the smoke from these fires, and millions of people were exposed to quantities of wildfire-generated particulate matter (PM) greatly in excess of the current PM standards. Hourly levels of PM with mass median aerodynamic diameter <2.5 μm (PM_2.5_) at Tracy (near our sampling site) peaked at 160 μg/m^3^ (San Joaquin Valley Air Pollution Control District 2008), whereas hourly concentrations of PM with mass median aerodynamic diameter < 10 μm (PM_10_) peaked at 200 μg/m^3^. Further to the north in the Sacramento River Valley, closer to the major fires, PM_2.5_ values of 262 μg/m^3^ were reported on the same days. Thus, PM with mass median aerodynamic diameter > 2.5 μm to < 10 μm (PM_10–2.5_) and PM_2.5_ concentrations were greatly in excess of the California 24-hr average ambient air quality standards (PM_10–2.5_, 50 μg/m^3^; PM_2.5_, 35 μg/m^3^) and among the highest values reported at these stations since data have been collected for PM pollution in these size classifications. These observations raise concerns about the potential health impact of exposure to high levels of wildfire PM, as the possible health effects associated with these acute exposures to PM from wildfires at these very high levels are not understood.

PM_10–2.5_ and PM_2.5_ samples were obtained during the last week of June 2008, when the fires were at their worst, from a U.S. Environmental Protection Agency (EPA) designated National Air Emissions Monitoring Study site that was heavily impacted. The monitoring included data on PM_10–2.5_ concentrations logged every 2 min (Series FH 62C14 Beta Sampler; Thermo Electron Corp., Franklin, MA). Peak value observed during the 2 days studied was 381 μg/m^3^, with values between 200 and 380 μg/m^3^ logged routinely over a period of several hours in the late afternoon and early evening of 26 June. Thus, the values reported at Tracy, the nearest San Joaquin Valley Air Pollution Control District monitoring site, probably underestimate the actual concentrations at our sampling site. This manuscript describes a toxicologic analysis of both the coarse and fine particles (PM_10–2.5_ and PM_2.5_) collected during the 2-day period of peak air pollution during June 2008, and compares the toxicity of wildfire PM with PM collected from nearby ambient air under normal conditions during June 2007. This manuscript will demonstrate that the inherent toxicity on an equal-dose basis is greater for the wildfire PM than that of PM from normal ambient air in this region. This is a novel and unexpected observation.

## Materials and Methods

Particulate matter used in this study was collected with a high-volume air sampler (model GS2310; Andersen Instruments Inc., Smyrna, GA) equipped with a four-stage cascade impactor (series 230, Andersen Instruments Inc.) in the summer months from a location in the northeast of the San Joaquin Valley in California. Slotted aluminum substrates (Tisch Environmental, Cleves, OH) were used for PM collection. The nominal flow rate used for collection was 20 ft^3^/min, with particle size cutoffs of 10.2, 4.2, 2.1, and 1.3 μm. For the purposes of this manuscript, we will refer to coarse PM as particles with a mass median aerodynamic diameter range of 10.2–2.1 μm and fine PM as particles within 2.1–1.3 μm. After collection, substrates from each stage were weighed; particles were removed by scraping with a spatula and stored at −80°C in vials. Thirty minutes before use, particles were suspended in phosphate-buffered saline (PBS), pH 7.6 (Mediatech, Inc., Herndon, VA) at the desired concentration. The final pH of the resultant suspension was pH > 7.

Bioassay techniques in the mouse have been validated and optimized as described previously (Wegesser and Last 2008). Briefly, male BALB/c mice 8–10 weeks of age (25–30 g) were purchased from Charles River Breeding Laboratories (Wilmington, MA). Mice were housed, four animals per cage, in filtered Bio-Clean facilities in the Animal Resources Center (University of California Davis, CA). Animals received water and standard feed (Purina Rat Chow) *ad libitum* and were allowed to acclimate for 1 week before any experimental procedures. The animals were kept on a 12-hr light/dark cycle at room temperature (68–70°F) and 30–70% relative humidity. All procedures were performed under an Institutional Animal Care and Use Committee approved protocol. All animals used in this study were treated humanely and with regard for alleviation of suffering.

Methods for intratracheal instillation of 50-μL suspensions of known amounts of PM into mice and evaluation of lung inflammation are described in detail elsewhere (Wegesser and Last 2008). Bronchoalveolar lavage (BAL) and tissue were collected 24 hr after PM instillation. Whole cell counts were performed with whole lavage fluid and a hemocytometer. Cells were separated from supernatant by centrifugation at 2,000 rpm in a benchtop centrifuge and stained with Diff-Quick (Fisher Scientific; Kalamazoo, MI) for differential cell counts. Protein content of lavage fluid supernatant was determined by a colorimetric reaction with the Micro BCA Protein Assay Reagent Kit (Pierce Biotechnology, Rockford, IL). Lavaged lungs were fixed at 30 cm pressure with 1% paraformaldehyde in PBS for 1 hr for histopathologic assessment, after staining with Harris’ hematoxylin and eosin, with an Olympus BH2 microscope connected to an OLY-750 Color Camera (Olympus; Center Valley, PA). Endotoxin in PM preparations was assayed by the Limulus amoebocyte lysate (LAL) assay (Wegesser and Last 2008).

Statistical analysis of data was performed with Prism 4.0 and 5.0 (GraphPad Software, San Diego, CA). All values are expressed as mean ± SE. Parametric analysis of data was conducted using analysis of variance with Tukey’s post-test for multiple comparisons. Differences were considered significant if the *p*-value (two-tailed) was < 0.05. Welch’s correction was applied if variances were found to be unequal.

## Results

**Figure 1:**
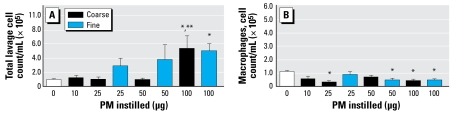
Number (mean ± SE) of total cells (*A*) and macrophages (*B*) recovered in lung lavage fluid from mice intratracheally instilled with different doses of PM_10–2.5_ (coarse) or PM_2.5_ (fine) from the wildfire samples (PBS-instilled controls, 0 μg). Note difference in *y*-axis scale in *A* and *B*. *p <0.001 compared with control. **p < 0.01 compared with 25 or 50 μg.

We found no significant differences in total cells recovered by lung lavage between either untreated (data not shown) or saline-instilled controls and mice instilled with 10, 25, or 50 μg wildfire PM_10–2.5_ (Figure 1A). There was a significant increase in total lavageable cells with instillation of 100 μg PM_10–2.5_ from the wildfire sample. In prior studies we have seen significant dose-related increases (more than twice as many cells) in total lavageable cells from mice instilled with 25 or 50 μg PM_10–2.5_ from ambient air samples collected from this geographic area (Wegesser and Last 2008), so the lack of increase in total lavageable cells seen between 10 and 50 μg PM_10–2.5_ from the wildfire samples is unusual.

**Figure 2:**
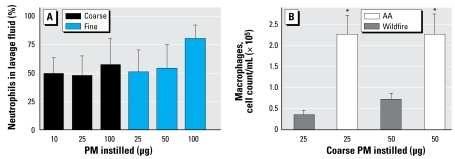
(*A*) Percentage of neutrophils in the lung lavage fluid from mice instilled with the indicated amounts of PM_10–2.5_ or PM_2.5_ wildfire PM. All of the indicated values are significantly greater than PBS-instilled controls, which contained 0% polymorphonuclear leukocytes. (*B*) Number of macrophages in the lung lavage fluid of mice instilled with either 25 or 50 μg PM: comparison of wildfire PM and normal AA PM collected 1 year earlier from the same area. Values shown are mean ± SE. *p < 0.05 compared with either 25 or 50 μg wildfire PM samples.

The cells lavaged from lungs of control mice were 95–100% macrophages, whereas lavage fluid from mice instilled 24 hr earlier with 50 μg PM_10–2.5_ from ambient air contained about 30% macrophages and 70% neutrophils (Wegesser and Last 2008). We found 49 ± 15, 47 ± 18, and 57 ± 23% neutrophils for mice instilled with 10, 25, or 100 μg wildfire PM_10–2.5_, respectively (Figure 2A). Thus, despite the lack of apparent increase in total cell numbers in the lung lavage from the mice exposed to 10 or 25 μg PM_10–2.5_ in Figure 1A, the mice responded to the wildfire PM at the lowest and the highest doses tested. The cell populations had shifted to about half neutrophils, which is not normal, despite total cell numbers remaining more or less constant. On an equal-dose basis, the wildfire lavage samples contained significantly lower numbers of macrophages than did lavage fluid from mice instilled with PM_10–2.5_ collected from normal ambient air (AA) during the same period 1 year earlier (Figure 2B; compare the responses to 25 and 50 μg wildfire PM_10–2.5_ with 25 AA and 50 AA, where 25 AA and 50 AA signify the samples of 25 and 50 μg PM_10–2.5_ from normal ambient air). Direct LAL assay shows < 1 endotoxin unit (EU) of endotoxin/50 μg PM_10–2.5_ preparation, ruling out a significant role for lipopolysaccharide (LPS) in the generation of the observed neutrophilic inflammation, as Balb/C mice respond normally to endotoxin (Silvia and Urosevic 1999).

The lung inflammatory response to PM_10–2.5_ from the wildfire differs from the response to PM_10–2.5_ from ambient air. Because the total number of lavageable cells did not increase in the mice exposed to 10–50 μg PM_10–2.5_ (Figure 1A), and half of the total cells were neutrophils, the wildfire PM_10–2.5_ must have caused a decrease in the numbers of macrophages in the lungs (Figure 1B). Note also that in the 100 μg wildfire PM_10–2.5_ sample, the total cells in the lavage were significantly increased and this increase was made up primarily of neutrophils (57% of the total cells). When compared with the 25 μg and 50 μg samples from normal AA, all animals dosed with PM_10–2.5_ from the wildfire (10, 25, 50, and 100 μg groups) had significantly fewer macrophages in their lung lavage fluid (Figure 2B). Thus, the most striking aspect of the cell differential counts in the mice exposed to the wildfire-derived PM_10–2.5_ is the relative absence of alveolar macrophages in their lungs, compared with PBS-instilled controls (Figure 2B). This accounts for the lower total cell count in these wildfire PM-exposed lungs, suggesting that either the wildfire PM_10–2.5_ may be especially toxic to pulmonary alveolar macrophages or that these wildfire PM_10–2.5_ create a condition in which macrophages are difficult to extract from the lungs by lavage, perhaps because of enhanced adherence to alveolar surfaces.

**Figure 3:**
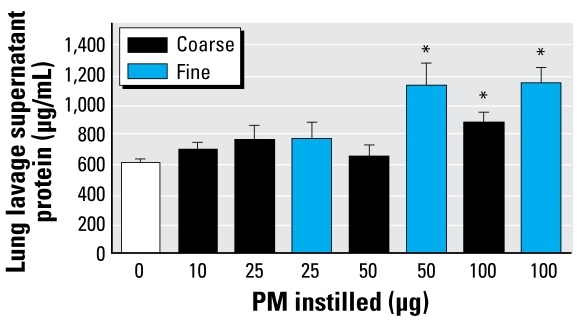
Protein content of lung lavage fluid supernatant of mice instilled with the indicated amounts of wildfire PM_10–2.5_ or PM_2.5_. *p < 0.05 compared with control.

As shown in Figure 3, there was significantly more protein in the lavage fluid supernatant from mice instilled with 100 μg of the PM_10–2.5_ fraction, and a trend toward higher amounts of protein in all of the groups examined compared with controls. The lack of a clear dose–response relationship in the data suggests that there is either a threshold in the observed response or that the lavage supernatant protein content is measuring a phenomenon more complex than simple fluid transudation across the airway epithelial barrier in a damaged lung (Witschi and Last 2001). In contrast, we have not observed any significant increase in lung lavage supernatant content of protein in mice exposed to PM_10–2.5_ preparations from normal AA collected from the San Joaquin Valley.

We performed similar experiments with PM_2.5_ preparations from the wildfire samples (Figures 1 and 3) collected simultaneously to facilitate direct comparisons of the two size fractions. We found significantly more total cells in the mice instilled with 100 μg wildfire PM_2.5_, with an apparent trend for dose response between 25 and 100 μg PM_2.5_ (Figure 1A). We observed significantly fewer macrophages in the lung lavage fluid from mice instilled with either 50 or 100 μg wildfire PM_2.5_ and comparable decreases of macrophages in mice instilled with 100 μg wild-fire PM_10–2.5_ or PM_2.5_ (Figure 1B). Both the 50 μg and 100 μg samples caused significant increases in the concentration of lung lavage supernatant protein in mice exposed to wildfire PM_2.5_ preparations (Figure 3), with an apparent dose-related difference in response to the 25 μg dose versus the 50- and 100-μg doses of PM_2.5_ tested. The increase in amount of protein in the lung lavage supernatant was not significantly different between the mice instilled with 100 μg PM_10–2.5_ or PM_2.5_.

**Figure 4:**
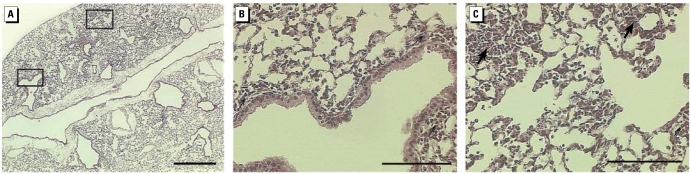
Representative lung sections from mice instilled 24 hr with 100 μg wildfire PM_10–2.5_. (*A*) Whole lung; bar = 500 μm. Boxes indicate areas shown in higher magnification in (*B*) and (*C*). (*B*) Proximal lung with conducting airways; bar = 100 μm. (*C*) Distal lung with centriacinar region; bar = 100 μm. Arrows indicate typical areas with inflammatory cell infiltrates. Sections from control animals are shown in Figure 5.

**Figure 5:**
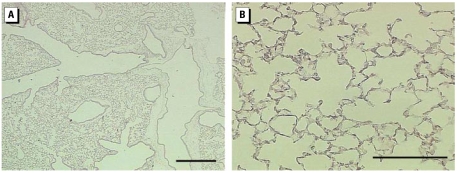
Representative lung sections from control mice instilled 24 hr with 50 μL PBS solution. (*A* ) Whole lung (low magnification) showing airways, blood vessels, and parenchyma; bar = 500 μm. (*B*) Lung parenchyma (high magnification) showing thin delicate alveolar septal tissues; bar = 100 μm.

**Figure 6:**
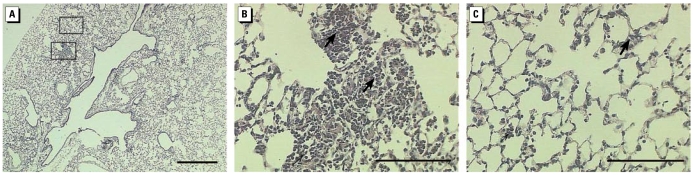
Representative lung sections from mice instilled 24 hr with 100 μg wildfire PM_2.5_. (*A* ) Whole lung (low-magnification; bar = 500 μm); boxes indicate areas shown in higher magnification in (*B*) and (*C*). (*B*) Centriacinar lung region showing the prominent accumulation of numerous inflammatory cells within alveolar airspaces. (*C*) Distal alveolar region with a diffuse increase in septal cellularity and occasional inflammatory cells within the alveolar airspaces. Arrows indicate areas of inflammatory cell influx. Bar = 100 μm in (*B*) and (*C*).

**Figure 7:**
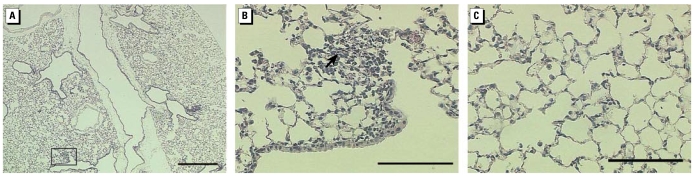
Representative lung sections from mice instilled 24 hr with 10 μg wildfire PM_10–2.5_. (*A*) Whole lung (low-magnification; bar = 500 μm); box indicates area shown in higher magnification in (*B*) and (*C*). (*B*) Centriacinar region with accumulations of inflammatory cells in the alveolar airspaces; arrow indicates area of cellular influx. (*C*) Distal alveolar region with subtle markings of pulmonary edema and increased abundance of alveolar macrophages. Bar = 100 μm in (*B*) and(*C*).

As shown in Figure 4, a marked influx of cells composed of monocytes and neutrophils was observed in mice instilled with 100 μg wildfire PM_10–2.5_ within the peribronchial tissues of the airways, along with an increased cellularity of septal tissues in the lung parenchyma with notable accumulation of inflammatory cells in the centriacinar airspaces of the lungs. Occasional extravasation of red blood cells, along with patchy edema fluid, was also noted in the alveolar airspaces. Increased lung tissue damage was noted with increasing doses of instilled particles for both PM_2.5_ and PM_10–2.5_ (Figures 4–7). In addition, wildfire PM_10–2.5_ particles induced greater histologic changes to the lungs for both the airways and the alveoli when compared with PM_10–2.5_ particles collected under normal ambient conditions.

## Discussion

There exists extensive literature on epidemiologic studies and a much smaller literature on whole-animal studies of the heath effects of exposure to woodsmoke from stoves, agricultural burning, wildfires, and other sources (Naeher et al. 2007; Zelikoff et al. 2002). Dubick et al. (2002) presented evidence of oxidative stress (lipid peroxidation) in lungs of rats acutely exposed (16 min) to whole woodsmoke by inhalation. Li et al. (1997) intratracheally instilled PM_10–2.5_ collected from AA in Scotland into rats and observed neutrophilic inflammation, increase in protein content, and oxidant stress (less glutathione) in lung lavage fluid from these animals, similar to our findings in this study. Many authors have examined the toxicity and proinflammatory activity of PM_10–2.5_ and/or PM_2.5_ by examination of their effects on cultured cells *in vitro* (e.g., Jiménez et al. 2002; Monn and Becker 1999; Veranth et al. 2004). Others have examined the toxicity of fractionated PM components to cultured cells (e.g., Adamson et al. 1999; Carter et al. 1997; Imrich et al. 2000). Specific toxicologic studies with PM isolated from wildfire smoke (e.g., Leonard et al. 2000; Jalava et al. 2006) are rare in the literature, presumably because of difficulty in collecting such PM fractions.

The lungs of mice exposed to wildfire PM_10–2.5_ or PM_2.5_ in the present study showed significant damage, as measured by histologic evaluation of inflammatory cell influx or by relative neutrophil content or total protein content of lung lavage fluid, compared with mice exposed to 10-fold higher doses of normal AA PM from the same area. The relative toxicity of PM_10–2.5_ and PM_2.5_ seemed similar in these experiments, but we should note that use of the intratracheal instillation route would mask differences in actual PM dosage to the lung of these different size fractions when they were inhaled. Based on the responses of mice to the 10 μg dose of wildfire PM_10–2.5_ or PM_2.5_ compared with the response to 50–100 μg PM from normal AA, we can estimate the relative toxicity of the wildfire PM on an equal-dose basis as about 10-fold more damaging than normal PM. Based on daily average PM mass collected with our high-volume sampler, there was about 2.2 times more PM_10–2.5_ and about 3.4 times more PM_2.5_ concentration in the air than on normal days in the region. Thus, a mouse exposed to the smoke-laden air from the wildfires would have been exposed to a relative risk of lung inflammation on the order of > 30 times the risk of breathing ordinary air in this region, which has some of the highest reported concentrations of PM_2.5_ in ambient air in the United States. In addition, the underlying mechanisms of toxicity may differ for the wildfire and for normal PM. The severity of the actual damage is masked during routine analysis of lung lavage parameters (cellularity or protein content) by the concomitant killing of pulmonary alveolar macrophages by the wildfire particles. The extent of damage to the lungs cannot be appreciated by *in vitro* analyses of PM_10–2.5_ or PM_2.5_ in cultured cells because of participation of extravasated blood and influx of inflammatory cells and edema fluid into the lung during pathologic changes. These observations highlight the critical importance of bioassays of toxicity of inhaled pollutants in whole animals as a component of a balanced scientific approach to estimating their toxicity.

Preliminary experiments suggest that active pro-inflammatory agent(s) in the wild-fire PM_10–2.5_ fraction is heat labile and extractable into an organic solvent, suggesting its organic nature. This is a reasonable hypothesis, given that the genesis of wildfire PM is from the incomplete combustion of biomass at relatively low temperatures. Others have suggested that aromatic chemical compounds, which can redox cycle, in PM derived from diesel exhaust or AA are able to damage lung cells and organelles by oxidative stress and are responsible for PM toxicity (Goldsmith et al. 1997; Laks et al. 2008; Xia et al. 2004). Consistent with this suggestion, we found dose-related increased staining for nitrotyrosine in the lungs of mice instilled with the wildfire PM_10–2.5_ but not the wildfire PM_2.5_. The active pro-inflammatory agent(s) in the wildfire PM_2.5_ need not be the same agent(s) responsible for the activity of the PM_10–2.5_. Studies with PM_10–2.5_ and PM_2.5_ collected from Alaska wildfire sites also implicate oxidative stress, in this case derived from free radicals arising (at least in part) from reactive metals in particles (PM_10–2.5_ and PM_2.5_), as a major source of carbon-centered free radicals responsible for their toxicity (Leonard et al. 2007).

Pulmonary alveolar macrophages may be a preferred target for PM toxicity, which affects macrophage function and specifically suppresses nitric oxide production by the macrophages (Antonini et al. 2002). Based on our results, there was no striking difference in the toxicity of the PM_2.5_ and PM_10–2.5_ fractions from the wildfire. PM_10–2.5_ was reported to have greater toxicity than PM_2.5_ from other sources (Kleinman et al. 2003), but our results suggest that relative toxicity of the two PM sizes may be assay dependent.

The use of intratracheal instillation as an exposure route may be criticized as unphysiologic because of the delivery of a bolus dose rather than a more gradual dose by inhalation exposure (Witschi and Last 2001). However, recent studies (Costa et al. 2006) suggest that if the total dose of PM instilled intratracheally remains in the physiologic range (i.e., equivalent to total dose achieved by acute inhalation exposure), then responses of laboratory animals to intratracheal administration are comparable with results found after inhalation exposure. Thus, intratracheal injection is an acceptable experimental approach to studying PM toxicity in whole animals.

Due to the sporadic and unpredictable nature of wildfires and the tendency for air pollution monitors to be situated in predominantly urban areas where population is concentrated, there has been relatively little systematic study of the toxicity of PM from wildfires in the literature. Hänninen et al. (2008) estimated population exposures in southern Finland to wildfire PM from a series of fires in Russia and the former Soviet Union in 2002. Their article reviews the existing epidemiologic data on exposures to PM from woodsmoke combustion, including wildfires, and concludes that within a large range of uncertainty, the effect of wildfire PM seems to be consistent with the effects of similarly sized PM from other sources of urban PM on an equal-exposure basis. However, total PM mass is higher during the wildfire episodes, so total toxicity would be greater. This conclusion is consistent with regulatory guidance from the World Health Organization and the U.S. EPA, where all PM of a given size class are assumed to be equally toxic regardless of source or chemical composition. Cell culture assays of wildfire-derived PM in mouse macrophages (Jalava et al. 2006) suggest that these size-fractionated PM preparations elicit similar or lesser toxicity on an equal-mass basis than normal ambient PM from the same sources. However, our comparative results testing PM in mice from normal AA and from AA during the wildfire suggest that the assumption that all particles of a given size class in the AA have the same toxicity (which is the basis for regulation of PM in the atmosphere) is an oversimplification.

We can conclude from these studies that the lungs of mice exposed to wildfire PM_10–2.5_ or PM_2.5_ show significant damage, as measured by histologic evaluation of inflammatory cell influx or by relative neutrophil or total protein content of lung lavage fluid, compared with mice exposed to 10-fold higher doses of normal AA PM from the same area. Thus, the inherent toxicity on an equal-dose basis is greater for the wildfire PM than that of PM from normal AA in this region. This is a novel and unexpected observation. Thus, a mouse exposed to the smoke-laden air from the wild-fires with peak hourly PM_10–2.5_ and PM_2.5_ concentrations about three times higher than normal peak PM concentrations in AA in this region, would be exposed to a relative risk on the order of > 30 times the risk of breathing ordinary air in the region. The relative toxicity of the PM_10–2.5_ and PM_2.5_ seemed to be similar in these experiments, but we should note that use of intratracheal instillation route would mask differences in actual PM dosage to the lung of these different size fractions on inhalation. Our observations in mice suggest that further research is required to test the assumption that all particles of a given size class in the ambient air have the same toxicity, the current regulatory approach paradigm.
